# Reliable prediction of implant size and axial alignment in AI-based 3D preoperative planning for total knee arthroplasty

**DOI:** 10.1038/s41598-024-67276-3

**Published:** 2024-07-23

**Authors:** Qing Lan, Shulin Li, Jiahao Zhang, Huiling Guo, Laipeng Yan, Faqiang Tang

**Affiliations:** 1 Shengli Clinical Medical College of Fujian Medical University, Fuzhou, China; 2 Department of Orthopedics, Fujian Provincial Hospital, Fuzhou, China; 3 Fuzhou University Affiliated Provincial Hospital, Fuzhou, China

**Keywords:** Medical research, Signs and symptoms

## Abstract

The size and axial alignment of prostheses, when planned during total knee replacement (TKA) are critical for recovery of knee function and improvement of knee pain symptoms. This research aims to study the effect of artificial intelligence (AI)-based preoperative three dimensional (3D) planning technology on prosthesis size and axial alignment planning in TKA, and to compare its advantages with two dimensional (2D) X-ray template measurement technology. A total of 60 patients with knee osteoarthritis (KOA) who underwent TKA for the first time were included in the AI (n = 30) and 2D (n = 30) groups. The preoperative and postoperative prosthesis size, femoral valgus correction angle (VCA) and hip-knee-ankle angle (HKA) were recorded and compared between the two groups. The results of the University of Western Ontario and McMaster University Osteoarthritis Index (WOMAC) and the American Knee Association Score (AKS) were evaluated before surgery, 3 months, 6 months, and 12 months after surgery. The accuracy of prosthesis size, VCA and HKA prediction in AI group was significantly higher than that in 2D group (*P* < 0.05). The WOMAC and AKS scores in AI group at 3 months, 6 months and 12 months after surgery were better than those in 2D group (*P* < 0.05). Both groups showed significant improvement in WOMAC and AKS scores at 12 months follow-up. AI-based preoperative 3D planning technique has more reliable planning effect for prosthesis size and axial alignment in TKA.

## Introduction

Total knee arthroplasty (TKA) is currently an effective method for the treatment of end-stage osteoarthritis. The purpose of TKA is to improve the patient's knee joint function and relieve pain symptoms by restoring the patient's normal knee joint biomechanical structure and rebuilding the lower limb force line^1^. The size and axial alignment of the knee prosthesis play decisive roles in these outcomes. However, an inappropriate knee prosthesis size and inaccurate axial alignment may lead to pain, a shortened prosthesis life, joint stiffness and joint instability in patients after TKA^2^. Therefore, the accuracy of preoperative TKA planning is crucial to the success of surgery.

Currently, the TKA preoperative planning techniques used in clinical practice can be divided into four main categories: 2D X-ray template measurement technology, patient-specific instrumentation technology (PSI) technology, robot assisted planning technology, and AI 3D planning technology. First, 2D X-ray template measurement technology, which is widely used because of its simple measurement and low cost. However, its accuracy is limited by differences in X-ray position, X-ray magnification, and empirical errors among different evaluators^3^. Second, although PSI technology can provide personalized planning models and guide the placement of osteotomy and prosthesis during surgery, but its high planning cost, lack of contribution to reducing surgical time^4^, and unstable planning accuracy^5^ limit its further promotion. Third, robot-assisted planning technology, relying on robot-assisted systems, can achieve accurate planning and implementation of relevant surgical steps^6,7^, but some studies have shown that robot-assisted TKA and traditional TKA do not have advantages in terms of the postoperative benefits for patients^8–11^.

AI-based preoperative 3D planning technology has shown more reliable prosthesis prediction accuracy than traditional technology in total hip replacement^12,13^, however, relatively few studies have reported the application of this technology in TKA. Therefore, the author reviewed and analysed the cases of 60 patients treated with TKA to examine the differences between AI-based preoperative 3D planning technology and traditional 2D template planning technology in terms of prosthesis size and axial alignment prediction and postoperative knee function.

## Materials and methods

### General information

The retrospective study was approved by the Ethics Committee of Fujian Provincial Hospital to retrospectively search our hospital database, and finally included 60 patients with KOA who received primary TKA at the Department of Orthopaedics of Fujian Provincial Hospital from September 2021 to June 2023. All operations were performed by the same surgical team via the midvastus approach. Among these patients, 30 patients underwent treatment with AI-based preoperative 3D planning technology (AI group), and 30 patients underwent treatment with the traditional 2D X-ray template measurement method (2D group). In the AI group, there were 10 males and 20 females, with a mean age of 69.10 ± 5.98 years and a mean BMI of 25.63 ± 3.00. In the 2D group, there were 8 males and 22 females, with a mean age of 69.37 ± 5.32 years and a mean BMI of 24.35 ± 3.98. This study meets the requirements of medical ethics and has been approved by the Ethics Committee of Fujian Provincial Hospital (K2022-08-035). All research conducted was performed in accordance with the relevant guidelines and regulations. As the study was a retrospective study without patient intrusion or intervention, all data were anonymized, so the author did not directly obtain consent from each patient. The need for informed consent was waived by the ethics committee of Fujian Provincial Hospital.

### Preoperative planning

**Figure 1:**
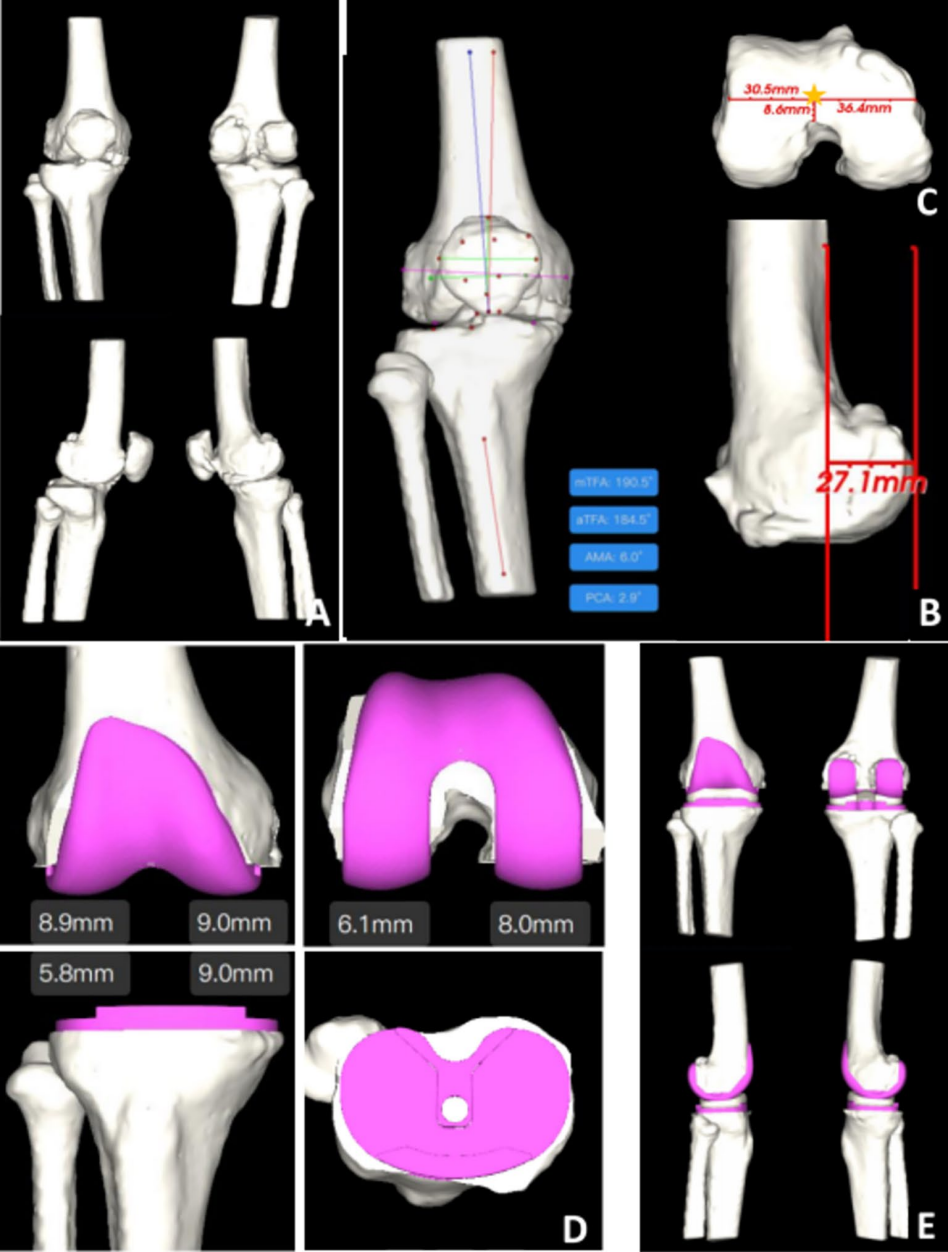
Schematic diagram of AI-KNEE 3D preoperative planning: (**A**) 3D reconstruction of knee joint; (**B**) automatically measure a range of knee joint parameters and measure posterior condylar offset; (**C**)intelligent prediction of the femoral opening point, asterisk is the predicted femoral opening point; (**D**) intelligent prediction of femoral and tibial prosthesis placement and simulation of prosthesis placement, automatic prediction of osteotomy volume; (**E**) simulated postoperative X-ray.

**Figure 2:**
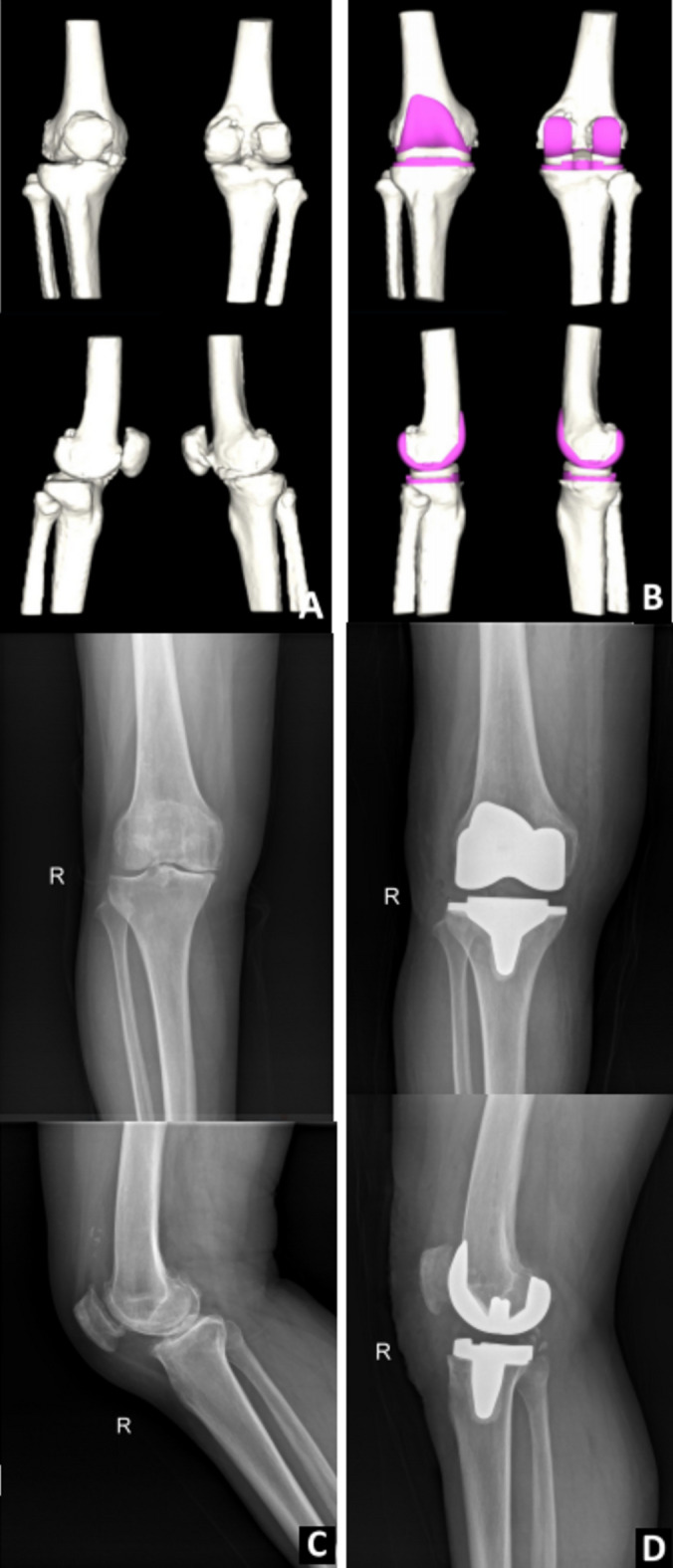
Schematic diagram of AI-KNEE 3D preoperative planning and postoperative imaging data: (**A**, **B**) AI 3D preoperative planning; (**C**) preoperative knee joint radiographs; (**D**) postoperative 3-day knee joint radiographs.

Patients in the AI group underwent plain computed tomography (CT) of the affected knee joint. The resulting CT imaging data in DICOM format and preoperative full-length imaging data of both lower limbs were anonymized and imported into the AI-Knee software system. By learning from many manually marked feature points and bone markers, the software automatically identifies relevant physiological and anatomical positions of the affected knee joint, automatically measures relevant parameters such as the axial alignment of the femur and tibia, and generates a 3D reconstruction of triaxial alignment (Fig. 1A). The software uses a unique self-developed algorithm (G-NET neural network) to identify the femur and tibia. The system calculates the extent of the tibial osteotomy according to the measured physiological back inclination of the tibia and the predicted back inclination of the osteotomy. The software then performs a simulated osteotomy of the tibia to predict and match the size of the tibial prosthesis. On the femoral side, the system marks the condylar line, the Whiteside line, and the femoral posterior condylar axis (Fig. 1B) on the 3D model to predict the femoral opening point (Fig. 1C). Then, the software accurately divides the femoral condyle according to the measured femoral valgus angle, etc., and predicts and matches the size of the femoral prosthesis via the same method (Fig. 1D). After the final matching, the final results of the AI preoperative planning and postoperative anteroposterior and lateral knee X-ray simulation are generated (Fig. 1E). The preoperative planning diagram and postoperative review comparison diagram of the AI group are shown in (Fig. 2A–D).

In the 2D group, on the basis of the anteroposterior and lateral images of the affected knee joint and the full-length weight-bearing radiographs, the 2D template was measured using the prosthetic film template provided by DePuy Synthes, and the optimal prosthesis size was selected according to the degree of matching between the prosthetic template and the femur and tibia.

### Operation technique

Patients in both groups were placed under general anaesthesia and in a supine position. Tourniquets were placed on the proximal thigh of the affected side before surgery, and routine disinfection was performed. Surgery via the midvastus approach was performed by the same surgical team in both groups. All knee prostheses used were from DePuy Synthes.

### Postoperative management and rehabilitation guidance

A drainage tube was opened in all patients 6 h after the operation and removed after 24 h or when the drainage volume was less than 50 ml. Postoperative analgesia was administered with an analgesic pump. Low-molecular-weight heparin was used for anticoagulation for 24 h after the procedure. After surgery, the affected limb was placed at 15° of abduction in a neutral position and a pillow was placed under the knee to reduce discomfort. The patients were guided in performing quadriceps functional exercises. After the drainage tube was removed, the patients were instructed to perform functional knee flexion and extension exercises. The patients were discharged upon meeting all of the following criteria: no significant knee swelling; no extension delay; active knee bending ≥ 90°; assisted walking distance ≥ 200 m; and visual analogue scale (VAS) pain score ≤ 4 points.

### Evaluation index

All demographic data and patient outcomes were collected by an independent trained surgeon. The evaluation indices included the femoral prosthesis size, tibial prosthesis size, postoperative VCA, HKA, preoperative Western Ontario and McMaster Universities Osteoarthritis Index (WOMAC) and American Knee Society (AKS) score and corresponding scores at 3 months, 6 months and 12 months after surgery.

### Statistical analysis

SPSS 22.0 statistical software was used to analyse the data. Measurement data conforming to a normal distribution are expressed as the mean ± standard deviation ($\overline{x} \pm s$) and were compared via T test. Count data are expressed as a rate (%) and were compared by chi-squared test. *P* < 0.05 was considered statistically significant.

### Informed consent

This study has been approved by the Ethics Committee of Fujian Provincial Hospital for research. Ethics number (K2022-08-035).

## Results

### Prosthesis fit and axial alignment accuracy

The surgery was successfully completed in all patients. Compared with the actual prosthesis size in the two groups, the rates of complete coincidence for the femoral and tibial prostheses in the AI group were 90% (27/30) and 86.7% (26/30), respectively, and those In the 2D group were 66.7% (20/30) and 60% (18/30), respectively, with a statistical significance (*P* < 0.05 for all), as shown in Table 1.

**Table 1:** Comparison of prosthesis models planned before operation between the two groups (n = 30).

Group	femoral prosthesis*					Tibial prosthesis*				
	− 2	− 1	0	+ 1	+ 2	− 2	− 1	0	+ 1	+ 2
AI group	0	1	27	2	0	0	2	26	2	0
2D group	0	4	20	3	3	0	2	18	6	4
P value	0.028					0.020				

*The condition of false body number conformity is divided into 5 grades of "− 2, − 1, 0, + 1, + 2", where "0" means full conformity.

" + " means that the size of the actual prosthesis used during surgery is larger than that planned before surgery, and "-" means that the size of the actual prosthesis used during surgery is smaller than that planned before surgery.

All patients underwent a full-length weight-bearing radiographs before surgery and 3 days after surgery. An imaging evaluation of the surgical effect was performed for all patients, and the postoperative VCA, defined as the angle between the mechanical and anatomical axes of the femur, of each patient was measured, calculated and compared with the preoperatively planned VCA. The HKA of each patient was also measured to evaluate the degree of deformity of the lower limb force line. The results are shown in Table 2.

**Table 2:** Comparison of imaging indicators between the two groups (n = 30, $\overline{x} \pm s$, %).

Group	Postoperative VCA	VCA outlier*	Postoperative HKA	HKA outlier*
AI group	6.06 ± 0.37	4(13.3%)	1.55 ± 0.82	1(3.3%)
2D group	5.63 ± 0.67	16(53.3%)	2.23 ± 1.35	9(30.0%)
P value	0.003	0.001	0.022	0.006

*VCA outlier: The difference between the predicted VCA before surgery and the actual VCA after surgery is ≥ 1°

*HKA outlier: The measurement angle exceeds neutral alignment angle (180°) by ≥ 3°

### Follow-up of knee joint function

All patients were followed up for 12 months. The WOMAC and AKS scoring systems were used to evaluate the remission of KOA and functional recovery of the knee joint, as shown in Table 3.

**Table 3:** Comparison of follow-up knee function scores (WOMAC scores AKS scores) between the two groups (n = 30, $\overline{x} \pm s$, points).

Group	Preop		PO 3 months		PO 6 months		PO 12 months	
	WOMAC	AKS	WOMAC	AKS	WOMAC	AKS	WOMAC	AKS
AI group	60.33 ± 7.14	46.63 ± 3.38	30.83 ± 2.11	63.35 ± 2.38	22.47 ± 1.99	83.03 ± 2.32	11.03 ± 2.25	93.07 ± 1.55
2D group	60.03 ± 4.25	46.10 ± 2.68	35.93 ± 1.38	59.90 ± 3.30	25.67 ± 2.05	80.58 ± 3.04	13.03 ± 2.56	91.47 ± 1.35
P value	0.844	0.501	< 0.001	< 0.001	< 0.001	0.001	0.002	< 0.001

## Discussion

Previous studies have shown that AI-based preoperative 3D planning technology has reliable accuracy in predicting the prosthesis size in total hip replacement^12,13^. Nevertheless, the efficacy of this technique in TKA is not clear, especially with respect to the prediction of the prosthesis size and axial alignment. Therefore, this study aimed to compare the efficacy of AI-based preoperative 3D planning technology and traditional 2D X-ray template planning technology in TKA.

In this study, the AI-KNEE was used for preoperative planning in patients with TKA. The complete coincidence rates of the femoral prosthesis and tibia prosthesis were 90% (27/30) and 86.7% (26/30), respectively, the outlier value of VCA was 13.3% (4/30), and the outlier value of HKA was 3.3% (1/30). Compared with the traditional 2D template planning technique, it more accurately predicts the prosthetic size and more reliably predicts postoperative lower limb alignment. Moreover, the postoperative follow-up patients also had better knee function scores.

The author reported that the accuracy of the prosthesis size prediction was significantly greater in the AI group than in the 2D group, with a complete coincidence rates for the femoral and tibial prostheses in the AI reconstruction group of 90% (27/30) and 86.7% (26/30), respectively. The corresponding rates in the 2D template group were 66.7% (20/30) and 60% (18/30), respectively, with good prediction accuracy. These rates of prosthesis prediction accuracy are significantly better than those reported by Miura and Kunze et al.^14–16^. The size of the prosthesis can greatly affect the outcome of TKA^17^. An excessively large prosthesis can lead to overfilling of the patella and excessive tension on the bilateral collateral ligaments, eventually leading to anterior knee pain, knee dysfunction and other adverse events. In contrast, a prosthesis that is too small will have a negative impact on knee flexion stability and postoperative knee pain, ultimately increasing the risk of prosthesis loosening^2^. Previously reported preoperative 3D planning software platforms, such as "Knee-PLAN", "Mimics" and "ZedKnee", offer the advantage of improving the accuracy of prosthesis size prediction; such software is usually based on preoperative CT 3D reconstructions and provides measurements and planning data through manual or AI systems. While these methods provide much more information than methods based on 2D X-ray templates, the long planning period and high technical threshold eventually became the factors limiting their further promotion^18^.

In the study, the outlier rates for the VCA and HKA in the AI group were 13.3% and 3.3%, respectively, which were significantly better than the 53.3% and 30.0% in the 2D group. In previous studies, only the prediction accuracy of the prosthesis size planned by the software was evaluated instead of comparing the effect on the axial alignment of the prosthesis at the same time. In the study, the VCA and HKA were selected to evaluate their effects on axial alignment. Although there is no consensus on which VCA is better for achieving a neutral lower limb force line, it is undeniable that the VCA plays a decisive role in the axial alignment of the lower limb. The study found that with decreasing VCA outliers, HKA outliers also decreased, indicating that the planned goal for a neutral lower limb force line could be better achieved with improved preoperative planning and prediction accuracy. Some studies have proposed that the measurement deviation of the VCA using 2D templates and preoperative 3D CT reconstructions is very small (0.15 ± 0.69°), but approximately 83.3% of the VCA difference is within 0–1°^19^. Interestingly, some studies have also shown that a personalized VCA may have advantages in improving limb alignment after surgery, especially in cases of severe knee deformity. In addition, the degree of matching between the personalized VCA and the fixed 4° VCA was less than 35.2%; however, the personalized VCA was less than 1° from the fixed 4° VCA in 68% of cases. Moreover, in Grade II cases of knee valgus deformity, an individualized VCA improved the alignment accuracy by 1° (1.8° ± 1.2° vs. 2.8° ± 1.5°, *p* = 0.00), indicating that VCA prediction accuracy has a significant impact on axial alignment, even when it is controlled within 1°^20^.

The AI group also showed satisfactory improvement in knee function after surgery. The knee function scores in the AI group were significantly better than those in the 2D group at 3 months, 6 months and 12 months after surgery. At present, there are few relevant data concerning knee joint function after AI-assisted TKA. Most studies have focused on developing AI programs to predict knee joint function after TKA, or using existing databases to verify the prediction accuracy of AI programs^21^. However, the significance of knee functional outcomes has not been fully determined between AI-assisted TKA and traditional TKA. Compared with that of the 2D group, the knee function score of the AI group was significantly improved 3 months after surgery, indicating that the AI preoperative assisted planning technology has advantages for short-term knee function improvement in TKA. The long-term follow-up of knee function after AI-assisted TKA should be further studied in order to better understand the postoperative benefits of AI-assisted technology for TKA.

The study had several limitations: (1) The implants used in the study were limited to devices from DePuy Synthes, and the study included a small range of implants. (2) The sample size of this study was small, and it was a single-centre retrospective study; thus, it is necessary to continue to expanding the number of cases or conduct randomized controlled trials in the future. (3) Relatively few parameters were evaluated. (4) The study only investigated the short-term and medium-term effects of preoperative AI reconstruction technology on the postoperative knee function of patients, and follow-up should be continued to observe and compare the long-term knee function of patients in the two groups.

## Conclusion

The use of AI 3D reconstruction technology for preoperative planning of patients to be treated with TKA can help clinicians more accurately plan the use of prosthesis size during surgery, and more reliably predict the postoperative VCA and HKA of patients to correct deformities associated with lower limb alignment, and ultimately improving the postoperative knee function score.

## Data Availability

The datasets generated during and/or analysed during the current study are not publicly available due to the protection of hospital and patient data but are available from the corresponding author on reasonable request.
